# Illness-Death Model as a Framework for Chronic Disease Burden Projection: Application to Mental Health Epidemiology

**DOI:** 10.3389/fepid.2022.903652

**Published:** 2022-06-27

**Authors:** Chisato Ito, Tobias Kurth, Bernhard T. Baune, Ralph Brinks

**Affiliations:** ^1^Institute of Public Health, Charité – Universitätsmedizin Berlin, Berlin, Germany; ^2^Department of Psychiatry, University of Münster, Münster, Germany; ^3^Department of Psychiatry, Melbourne Medical School, University of Melbourne, Melbourne, VIC, Australia; ^4^Florey Institute of Neuroscience and Mental Health, University of Melbourne, Melbourne, VIC, Australia; ^5^Medical Biometry and Epidemiology, Faculty of Health, School of Medicine, Witten/Herdecke University, Witten, Germany; ^6^Institute for Biometrics and Epidemiology, German Diabetes Center (DDZ), Leibniz Center for Diabetes Research, Heinrich Heine University, Düsseldorf, Germany

**Keywords:** burden of disease, illness-death model, epidemiological models, projection, prevalence, chronic disease, anxiety disorders, mental health

## Abstract

**Introduction:**

Estimates of future disease burden supports public health decision-making. Multistate modeling of chronic diseases is still limited despite a long history of mathematical modeling of diseases. We introduce a discrete time approach to the illness-death model and a recursion formula, which can be utilized to project chronic disease burden. We further illustrate an example of the technique applied to anxiety disorders in Germany.

**Materials and Equipment:**

The illness-death model is a multistate model that relates prevalence, incidence, mortality, and remission. A basic recursion formula that considers prevalence, incidence, mortality among the susceptible, and mortality among the diseased can be applied to irreversible chronic diseases such as diabetes. Among several mental disorders, remission plays a key role and thus an extended recursion formula taking remission into account is derived.

**Methods:**

Using the Global Burden of Disease Study 2019 data and population projections from the Federal Statistical Office of Germany, a total number of individuals with anxiety disorders by sex in Germany from 2019 to 2030 was projected. Regression models were fitted to historical data for prevalence and incidence. Differential mortality risks were modeled based on empirical evidence. Remission was estimated from prevalence, incidence, and mortality, applying the extended recursion formula. Sex- and age-specific prevalence of 2019 was given as the initial value to estimate the total number of individuals with anxiety disorders for each year up to 2030. Projections were also made through simple extrapolation of prevalence for comparison.

**Results:**

From 2019 to 2030, we estimated a decrease of 52,114 (−1.3%) individuals with anxiety disorders among women, and an increase of 166,870 (+8.5%) cases among men, through the illness-death model approach. With prevalence extrapolation, an increase of 381,770 (+9.7%) among women and an increase of 272,446 (+13.9%) among men were estimated.

**Discussion:**

Application of the illness-death model with discrete time steps is possible for both irreversible chronic diseases and diseases with possible remissions, such as anxiety disorders. The technique provides a framework for disease burden prediction. The example provided here can form a basis for running simulations under varying transition probabilities.

## Introduction

Understanding the current distribution and burden of a disease in a population of interest is at the core of epidemiology. As the already immense burden of chronic diseases continues to grow, in addition to understanding the present-day situation, questions such as “what would be the burden of chronic disease A in X years?” becomes pertinent. Answers to these questions help us gain insight into the future and can inform public health interventions. While answering such a question is not a simple or easy task, using mathematical models and the best available data of the present day, it is possible to make reasonable projections under certain assumptions.

Mathematical modeling of diseases, particularly infectious diseases, has a long history, dating back to 1760 when Daniel Bernoulli showed the benefit of smallpox inoculations on life expectancy (1). Early conception of multistate modeling of communicable diseases is seen in the work of Kermack and McKendrick, which is the basis of the Susceptible-Infectious-Removed (SIR) model (2, 3). Although relatively later, applications of multistate modeling to non-communicable diseases started to appear in the mid-twentieth century (4).

**Figure 1:**
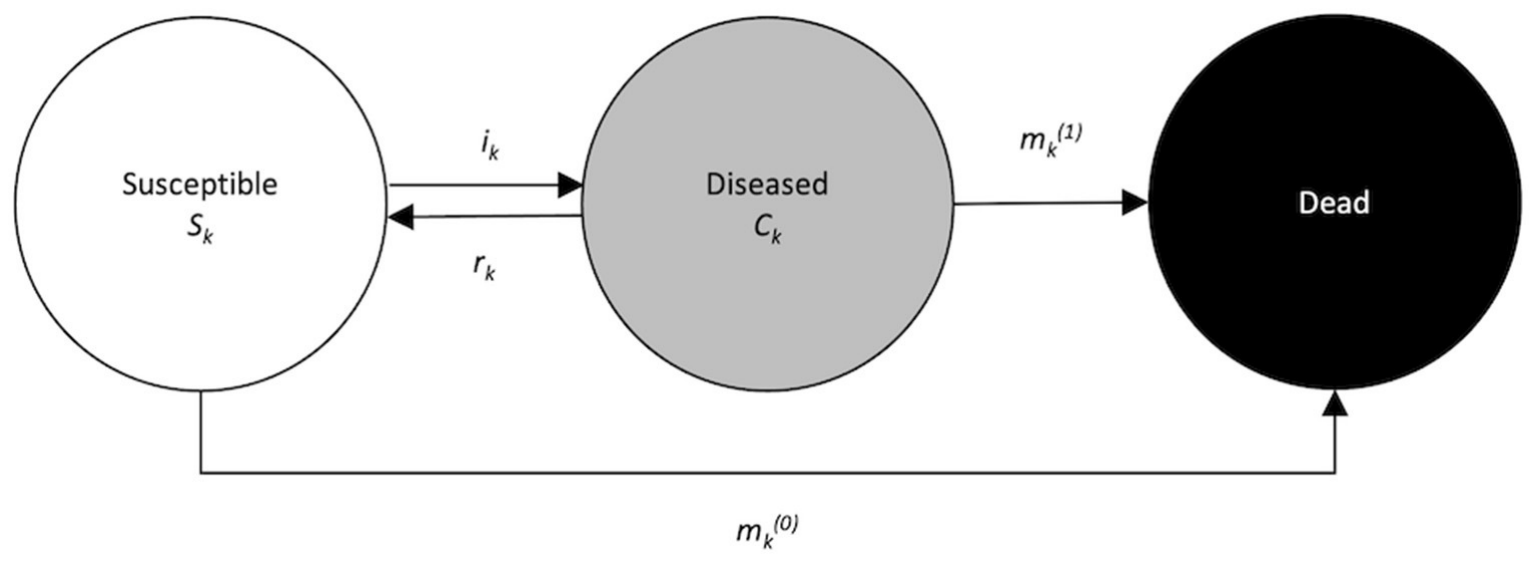
Illness-death model for chronic diseases with remission. The population under consideration is divided into three disjunct compartments: susceptible (with respect to the disease of interest), diseased, and dead. Arrows between the states indicate possible transitions.

In 1991, Keiding (5) related age-specific incidence and prevalence in a population by modeling individuals' dynamics between healthy, disease, and death states, bridging an individual-based statistical approach and a population-focused epidemiological approach. As shown by Keiding, in non-communicable disease modeling we are interested in individuals' transition in a population from a healthy or susceptible state to a diseased state, then to death either from the diseased state or directly from the susceptible state, represented in the illness-death model (Figure 1).

In more recent years, Brinks et al. (6) demonstrated that age-specific incidence rates can be estimated from cross-sectional prevalence data using a partial differential equation and extended its application to chronic disease epidemiology. Tönnies et al. (7) have applied this approach to project the age-specific prevalence of type 2 diabetes mellitus in Germany using health insurance claims data.

While disease modeling of chronic diseases may be gaining traction given its potential to help improve public health intervention planning, its application is still limited. Moreover, the approach based on a partial differential equation applied for diseases such as diabetes leaves a technical hurdle for practical application, as it requires the investigators to be familiar with the theory of differential equations. We think that the application of the illness-death model can be made more accessible to a broader range of practitioners by taking a discrete time approach; and that it can be extended to chronic diseases that are of increasing public health concern, for instance, psychiatric disorders, to better understand its population-level impacts over time.

Building on the previous non-communicable disease modeling efforts, in this paper we first aim to introduce a discrete time approach to the illness-death model and a recursion formula that relates prevalence, incidence, and mortality. Further, we present an extension of the recursion formula that considers chronic diseases with remission. Our second aim is to illustrate an application of the illness-death model in mental health epidemiology with anxiety disorders as an example to offer step-by-step guidance for researchers who are interested in applying the method to other areas.

## Materials and Equipment

The illness-death model is a multistate model, in which relationships between incidence, prevalence, and mortality can be leveraged to model population disease burden. Here we discuss a discrete time approach as it is a simple yet robust approach.

Using this technique, age- and/or period-specific prevalence-odds and prevalence of a disease at a point in time can be estimated when an incidence risk, mortality risk among the susceptible, and mortality risk among the diseased in a previous time point are given. Below we show the derivation of a recursion formula.

Population groups in different compartments of the illness-death model (Figure 1) and transition probabilities between states are denoted as follows:

*S*_*k*_ = Number of individuals who are susceptible to the disease, or without the disease at time *t*_*k*_.

*C*_*k*_ = Number of individuals who have the disease at time *t*_*k*_.

*i*_*k*_ = disease incidence (risk) during time (*t*_*k*_*, t*_*k*+1_].

$m_{k}^{\left(1\right)}$ = mortality risk among the diseased during time (*t*_*k*_*, t*_*k*+1_].

$m_{k}^{\left(0\right)}$ = mortality risk among the susceptible during time (*t*_*k*_*, t*_*k*+1_].

where time scale *t*_*k*_ = *k*_τ_ *with* τ > *0, k* = *0,1, ..*.

### Recursion Formula: Irreversible Chronic Diseases

By relating the number of susceptible individuals and the number of diseased individuals at *t*_*k*+1_to the preceding time point of *t*_*k*_, the number of susceptible individuals at time *t*_*k*+1_is expressed as a difference equation:


(1) $$\begin{array}{r} S_{k+1}=\;S_{k}-i_{k}\;S_{k}-m_{k}^{\left(0\right)}S_{k} \end{array}$$

Similarly, the number of diseased individuals at time *t*_*k*+1_is:


(2) $$\begin{array}{r} C_{k+1}=\;C_{k}+i_{k}\;S_{k}-m_{k}^{\left(1\right)}C_{k} \end{array}$$

Further, given that the prevalence of the disease at time *t*_*k*+1_is $p_{k+1}=\frac{C_{k+1}}{S_{k+1}+\;C_{k+1}\;}\;$, prevalence odds π at time *t*_*k*+1_ is:


$$\begin{array}{l} \pi_{k+1}\;=\frac{p_{k+1}}{1-p_{k+1}}\;=\frac{C_{k+1}}{S_{k+1}\;} \end{array}$$

Using the difference Equations 1, 2,


(3) $$\begin{array}{l} \begin{array}{c} \pi_{k+1}\;=\frac{C_{k}\;+i_{k}\;S_{k}-m_{k}^{\left(1\right)}C_{k}}{S_{k}\;-\;i_{k}\;S_{k}-m_{k}^{\left(0\right)}S_{k}\;}\\ \;=\frac{(1-\;m_{k}^{\left(1\right)})\frac{C_{k}}{S_{k}}+i_{k}}{1-\;i_{k}\;-m_{k}^{\left(0\right)}\;} \end{array}\\ \begin{array}{c} \;=\;\frac{(1-m_{k}^{\left(1\right)})\pi_{k}\;+\;i_{k}}{1\;-\;i_{k}-m_{k}^{\left(0\right)}} \end{array} \end{array}$$

recursion formula (Equation 3) is derived. Further details on this derivation are provided by Brinks and Hoyer (8). When a starting point π_0_ is given, subsequent values of the prevalence odds π_*k*_ at time *k* = *1,2,3…*, can be calculated using this recursion formula. This basic recursion formula can be applied to estimate the prevalence, incidence, or mortality of an irreversible non-communicable disease such as dementia and type 2 diabetes mellitus (9), depending on a measure of interest and data availability.

### Extension of Recursion Formula: Chronic Diseases With Remission

Many chronic diseases are irreversible, meaning that once an individual becomes diseased, they will remain in the diseased state until death, and will not move back into the susceptible state. Hence, the model accounting for transition probabilities including incidence risk, mortality risk among the diseased, and mortality risk among the susceptible is appropriate and sufficient to capture the dynamics of such a disease.

However, the basic recursion formula falls short of capturing an important dynamic of some chronic diseases, in which some individuals in the population move back and forth between the non-diseased and diseased states, or asymptomatic and symptomatic states. For example, for a subset of mental disorders, such as anxiety disorders, remission is possible and is a treatment goal (10). This motivates us to extend the recursion formula by addition of the remission probability *r*, where,


$$\begin{array}{l} r_{k}\;=\;remission\;probability\;among\;the\;diseased\;during\;time\\ \;(t_{k},t_{k+1}]. \end{array}$$

Note that “remission” probability may not always be the most appropriate term to reflect this phase in the life course of a certain disease, and therefore it may be termed differently depending on a clinical context. For instance, a risk of an exchange between active and non-active cases, rather than remission probability, may be a more appropriate description in certain settings. We use the term remission here to simply mean an individual's transition from the diseased state (i.e., cases) to the susceptible state (a backward arrow in Figure 1).

With an addition of the remission probability, the difference Equations 1, 2 are transformed to:


(4) $$\begin{array}{r} S_{k+1}=\;S_{k}-i_{k}\;S_{k}\;+r_{k}\;C_{k}-m_{k}^{\left(0\right)}S_{k} \end{array}$$

(5) $$\begin{array}{r} C_{k+1}=\;C_{k}+i_{k}\;S_{k}-r_{k}\;C_{k}-m_{k}^{\left(1\right)}C_{k} \end{array}$$

and thus, the prevalence-odds π at time *t*_*k*+1_ is formulated as:


(6) $$\begin{array}{l} \begin{array}{c} \pi_{k+1}\;=\frac{\;C_{k}+i_{k}\;S_{k}-r_{k}\;C_{k}-m_{k}^{\left(1\right)}C_{k}}{S_{k}-i_{k}\;S_{k}\;+r_{k}\;C_{k}-m_{k}^{\left(0\right)}S_{k}\;}\\ \;=\frac{(1-r_{k}\;-m_{k}^{\left(1\right)})\frac{C_{k}}{S_{k}}+i_{k}}{r_{k}\;\frac{C_{k}}{S_{k}}+(1-i_{k}\;-m_{k}^{\left(0\right)})\;} \end{array}\\ \;\begin{array}{c} =\;\frac{(1-r_{k}\;-m_{k}^{\left(1\right)})\pi_{k}+i_{k}}{r_{k}\pi_{k}+(1-i_{k}\;-m_{k}^{\left(0\right)})\;} \end{array} \end{array}$$

Equation 6 is the extended recursion formula that takes remission into account. Like the recursion formula (Equation 3), one can enumerate age- and/or period-specific prevalence-odds, or other transition probabilities in the formula depending on a question and data availability, by giving a starting value and recurrently applying the Equation 6. For *r*_k_ = 0, Equation 6 becomes Equation 3. Hence, Equation 6 is a generalization of Equation 3.

## Methods

In this section, we demonstrate an application of the extended recursion formula using an example from the mental health field. We focus specifically on anxiety disorders, which is one of the most globally prevalent, yet underestimated, mental disorders (11). It was estimated that there were 304.1 million cases of anxiety disorders globally in 2019 (12). While the evidence is limited, anxiety disorders tend to be chronic, and the duration is varying. Cumulative full remission probability among adult patients ranged from 0.31 to 0.76 over 8 years in anxiety disorders including social phobia, generalized anxiety disorder, panic disorder, and panic disorder with agoraphobia, in the Harvard/Brown Anxiety Research Program (13). A population-based study (14) found that its cohort's mean anxiety episode duration was 15.2 months and the median was 7.2 months in their 3-year follow-up, while one-third of the participants with an anxiety disorder had not recovered at 36 months. A specific aim here is to project the age-specific prevalence and therefore the total number of people with anxiety disorders by sex in Germany, from 2019 to 2030.

### Data Sources

Age- and sex-specific incidence rates and prevalence of anxiety disorders in Germany from 1990 to 2019 were obtained from the Global Burden of Disease Study 2019 (GBD) (15). The GBD provides disease burden estimates in terms of incidence, prevalence, mortality, years of life lost, as well as morbidity estimates such as disability-adjusted life-years and years lived with disability, for a total of 369 diseases and injuries for 204 countries and territories (16). In the GBD 2019, anxiety disorders were defined as a group of anxiety disorder subtypes, which corresponded to the International Statistical Classification of Diseases and Related Health Problems 10th Revision (ICD-10) codes F40, F41, F42, F43.0, F43.1, F93.0, F93.1, F93.2, and F93.8 (12). The GBD data set provided the baseline disease trends from 1990 to 2019 and starting values for our projection. To account for the expected changes in the population size over time, population data of Germany with a projection up to 2060 were obtained from the Federal Statistical Office of Germany (17). Both of these data sets are publicly available online (Supplementary Table 1).

### Procedures

First, we modeled prevalence, incidence rates, mortality risk among the diseased, and mortality risk among the susceptible based on historical data and empirical evidence.

#### Prevalence

For each sex, we fitted a regression model to the prevalence from the GBD data from 1990 to 2019 as a function of age *k* and year *j*.

#### Incidence

For each sex, we fitted a regression model to incidence rates from the GBD data from 1990 to 2019 as a function of age *k* and year *j*.

#### Mortality

We modeled mortality risk among the diseased and mortality risk among the susceptible differentially in terms of relative mortality risk, based on empirical evidence. We used the risk ratio of 1.4, a ratio of all-cause mortality risk among people with anxiety disorders to those without, as reported by Walker et al. in their meta-analysis of 29 studies (18). As the available evidence is not sex-specific, we used the same models for both sexes. Additionally, we assumed a gradual decrease in mortality risks over time both among people with anxiety disorders and those without, but a faster rate among the diseased, which can be expected through various medical advances. A greater decline in mortality rates over time among the diseased compared to those without has been observed in other chronic diseases, such as diabetes (19).

#### Remission

As data on remission were not available, we estimated remission probabilities across all ages (1–95 years) over the data period (1990–2019) using the extended recursion formula. Through algebraic manipulation and an addition of period-dependency *j*, Equation 6 can be formulated as follows to obtain the remission probability *r*_*j, k*_:


$$\begin{array}{l} r_{j,k}\;=\frac{\left(1-m_{j,k}^{\left(1\right)}\right)\pi_{j,k}\;-\left(1-m_{j,k}^{\left(0\right)}\right)\pi_{j+1,k+1}\;+\left(1+\pi_{j+1,k+1}\right)\;i_{j,k}}{\left(\pi_{j+1,k+1}\;+\;1\right)\pi_{j,k}\;} \end{array}$$

We then modeled remission probability as a function of age *k* and year *j*.

#### Projection

We set 2019–2030 as a time frame for our projection. Based on population projection estimates of the Federal Statistical Office of Germany (Destatis) (17), the sex-specific age distribution of the population in 2019 was used to give initial values, and the number of individuals entering the population each year until 2030 was set, assuming that births are uniformly distributed across each year. Out of the 27 variants of projections with varying combinations of assumptions that the Destatis data provide, we selected Variant 2 for our projection. Variant 2 assumes that: the fertility rate will stabilize at 1.55 children per woman; there will be a moderate increase in life expectancy at birth from 83.2 to 88.1 years among women and from 78.4 to 84.4 years among men by 2060, compared to the life table 2015/2017; and net migration (i.e., balance of immigration and migration) will decrease from 386,000 people per year in 2018 to 206,000 in 2026 and remain stable from there on (20). We assumed that the prevalence of anxiety disorders is similar among the German population and the migrants (both immigrants and emigrants). Brinks and Landwehr (9) previously showed that the change in prevalence does not depend on migration when the disease prevalence is the same among the original population and the migrant population; and that even when this differs between the two groups, the impact of migration on prevalence was relatively small.

By using the age-specific prevalence of the base year 2019 based on the fitted model, and all the fitted models for incidence rates, mortality among the diseased, mortality among the susceptible, as well as remission, and repeatedly applying difference Equations 4, 5, sex-, age-, and period-specific number of individuals with anxiety disorders and those without the disease up to 2030 were calculated. We used a time step of 1 week for the calculation. This means that, for example, a number of cases in the 2nd week of January 2019 is enumerated based on the value in the 1st week of January 2019. We then obtained the total number of individuals with and without anxiety disorders for each projection year among women and men, respectively, by summing all age-specific estimates in each year.

#### Projection With Prevalence Extrapolation

For the purpose of comparison, we also estimated the number of anxiety disorder cases in each year from 2019 to 2030 for each sex through simple extrapolation of the prevalence. This was done by extrapolating the regression model fitted to the baseline prevalence data from 1990 to 2019 to the projection time frame and multiplying the projected population for each year from 2019 to 2030. We used Variant 2 of the population projections, as we did with the illness-death model approach described in the previous section.

### Software

All analyses were performed using R (version 4.1.2) (21) and RStudio (version 2022.02.0+443) (22). We have made our R analysis codes publicly available (23).

## Results

**Figure 2:**
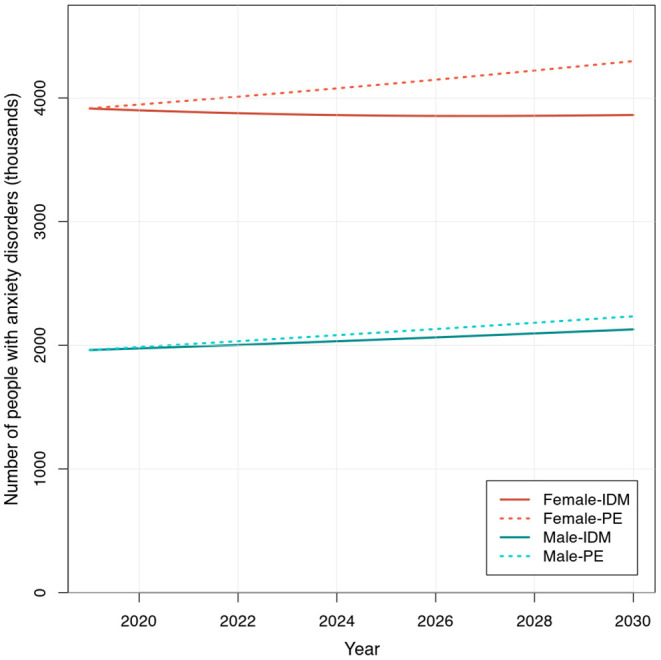
Projected number of people with anxiety disorders in Germany from 2019 to 2030. Number (in 1,000's) of male and female individuals with anxiety disorders, projected using the extended recursion formula of the illness-death model (IDM) and through prevalence extrapolation (PE), based on the Destatis population projection estimates (Variant 2) from 2019 to 2030.

Figure 2 shows the projected total number of people with anxiety disorders in Germany from 2019 to 2030 by sex, resulting from the illness-death model application and the simple extrapolation of prevalence. In 2019, there were an estimated 3.91 million women and 1.96 million men with anxiety disorders. Based on the illness-death model approach, among women, we forecasted a slight decrease of 52,114 (−1.3%) individuals with the disease from 2019 to 2030, resulting in an estimated total of 3.86 million women with anxiety disorders in 2030. In contrast, among men, we estimated an increase of 166,870 (+8.5%) cases over a decade, with a total expected number of 2.12 million men suffering from anxiety disorders in 2030.

Of note, in the process of estimating remission probabilities using the extended recursion formula, we observed a portion of 2,850 estimates (95 age groups in each of the 30 years from 1990 to 2019) taking values below 0 and >1 [for female: 294 out of 2,850 (10.3%); for male: 310 out of 2,850 (10.9%)]. Since remission probability must be 0 ≤ *r*_*j, k*_ ≤ 1, these values were excluded before we modeled the remission probability.

As a snapshot of transition probabilities used in the illness-death model approach, age-specific incidence, mortality, and remission probability among women in 2019 is shown in Supplementary Figure 1.

When we simply extrapolated the model fitted to prevalence from 1990 to 2019 using the projected population for each year from 2019 to 2030, the resulting estimates of people with anxiety disorders showed a different pattern from the results obtained through the illness-death model. With this approach, we saw a large increase of 381,770 (+9.7%) individuals with the disease in 2030 compared to 2019 among women, totaling 4.30 million cases in 2030. There was an even greater increase of 272,446 (+13.9%) cases observed among men, with a total of 2.23 million individuals with anxiety disorders in 2030. The comparison of estimates obtained by the illness-death model and the simple prevalence extrapolation, and changes from 2019 in case numbers and percentage for each sex are summarized in Tables 1, 2.

**Table 1:** Projected number of women with anxiety disorders in Germany from 2019 to 2030: comparison of illness-death model and simple prevalence extrapolation.

**Estimated number of people with anxiety disorders**						
**Female**						
	**Illness-death model**			**Extrapolation of prevalence**		
**Year**	**Cases (1,000's)**	**Change from 2019 (1,000's)**	**% change from 2019**	**Cases (1,000's)**	**Change from 2019 (1,000's)**	**% change from 2019**
2019	3,914.45	0.00	0.00	3,916.11	0.00	0.00
2020	3,900.15	−14.30	−0.37	3,946.96	30.85	0.79
2025	3,857.13	−57.31	−1.46	4,112.01	195.90	5.00
2030	3,862.33	−52.11	−1.33	4,297.88	381.77	9.75

**Table 2:** Projected number of men with anxiety disorders in Germany from 2019 to 2030: comparison of illness-death model and simple prevalence extrapolation.

**Estimated number of people with anxiety disorders**						
**Male**						
	**Illness-death model**			**Extrapolation of prevalence**		
**Year**	**Cases (1,000's)**	**Change from 2019 (1,000's)**	**% change from 2019**	**Cases (1,000's)**	**Change from 2019 (1,000's)**	**% change from 2019**
2019	1,961.54	0.00	0.00	1,961.86	0.00	0.00
2020	1,974.96	13.42	0.68	1,985.57	23.71	1.21
2025	2,048.05	86.51	4.41	2,106.45	144.59	7.37
2030	2,128.41	166.87	8.51	2,234.31	272.45	13.89

## Discussion

In this paper, our primary objective was to introduce a discrete time approach of the illness-death model and a recursion formula that relates prevalence, incidence, and mortality to each other, as well as its extension by an addition of remission. Our secondary objective was to illustrate an application of the extended recursion formula, by using anxiety disorders as an example and projecting prevalence and thus the total number of people with the disease in Germany until 2030.

As shown in our derivation of the recursion formulae, the illness-death model with discrete time steps can be applied to both irreversible chronic diseases and chronic diseases with a possibility of remission. For non-reversible chronic diseases, a similar method, though in a continuous time scale using a partial differential equation, has previously been applied to diseases such as type 2 diabetes mellitus and dementia (7, 9). The extension of the recursion formula with remission in the domain of anxiety disorders will allow for a better projection of this important mental health condition. This method can also be applied to other mental disorders in which remission plays a key role, as well as other non-communicable diseases such as cancer, although the utility is limited within the realm of chronic diseases, as the illness-death model does not model key elements for communicable diseases, such as the interaction between individuals and transmissions.

The recursion formula makes it possible to project disease burden even if population-level data are not available for all transition probabilities in the illness-death model. Since remission probability data were not available in our example, they were estimated using other measures available—incidence, prevalence, and mortality—through calculation using the extended recursion formula. In other words, estimation of a missing transition probability in the model can be achieved as long as estimates are available, or reasonable assumptions can be made for the remainder of the parameters. In terms of population projection data, we utilized data provided by the Federal Statistical Office of Germany in our example. Even if such population projections were not locally available at a specific country or region level of interest, this type of demographic data is made available by various bodies such as the United Nations and the World Bank (24, 25).

The number of people with anxiety disorders in Germany from 2019 to 2030 by sex, was estimated using the extended recursion formula. The projections based on the illness-death model approach indicated a slight decrease in the total number of cases of anxiety disorders among women and a large increase among men over the next decade. In contrast, when projections were made simply by extrapolating the historical trend in prevalence, a much larger increase in the case number was estimated both among men and women. The latter approach may be attractive for its apparent simpleness; however, this may, in fact, be an undesirable oversimplification. Extrapolation of a fixed prevalence onto future population projections ignores incidence, remission, and mortality, all of which together comprise disease prevalence in a population. Disregarding these measures and their interplay is to ignore complex disease dynamics aside from sheer changes in population distributions. An assumption that incidence, remission, and mortality do not change and thus prevalence would be constant over time rarely holds true, hence projections under such an assumption are less reliable. In a recent work by Voeltz et al. (26), a comparison was made between the type 2 diabetes case number projections obtained by a simple prevalence-based approach and by the illness-death model approaches using partial differential equations. Their results showed a stark difference in estimates, with the prevalence extrapolation leading to a much smaller estimated increase in the disease prevalence (in their three methods, the estimated increase from 2010 to 2040 ranged from +29% based on the simple prevalence-based approach, to +116% based on an illness-death model approach) (26). While the difference in estimates we observed in our analysis of anxiety disorders is not as dramatic, the change in the direction of trends seen among women (−1.3% decrease with the illness-death model vs. +9.7% increase with prevalence extrapolation in Table 1) highlights the possibility of arriving at quite different conclusions when disease-specific measures that make up prevalence are considered and when they are not. Models with unrealistic assumptions may inadequately project disease occurrence into the future and may mislead subsequent population intervention or resource allocation planning. We suggest the use of the illness-death model whenever possible, as it would also allow to anticipate different scenarios in which incidence, remission, and/or mortality suddenly change due to external factors. Reflecting changes in specific transition probabilities in projections is not possible when prevalence, a composite of these transition probabilities, is assumed to be constant. In case the input parameters have statistical uncertainties, we recommend using a multidimensional probabilistic resampling technique. The main idea is to randomly sample from the distributions of input parameters (i.e., incidence, mortality, and remission), combine the different input parameters, and calculate the relevant outcomes. If we repeat this resampling and recalculation, the distribution of the outcomes reflects the combined uncertainty based on the uncertainty in the input parameters. Details about this method can be found in Oakley and O'Hagan's work (27).

There are some limitations to this illness-death model approach. While there is an advantage of simplicity and relative ease of application with our approach, theoretically the accuracy of estimates is inferior to the continuous time approach using the partial differential equation (9). However, if the time step (i.e., the difference between *t*_*k*_ and *t*_*k*+1_) is set to be small, the difference in outcome estimates between the discrete and continuous time approach also becomes small (9). In mathematical terms, as this time increment approaches zero, the recursion formula converges to the differential equations. Details are shown in textbooks about modeling, e.g., Vynnycky and White (28). In the demonstrated example, we used a time step of 1 week, which is small enough to achieve a similar level of accuracy as the differential equations. Researchers who intend to use these tools should carefully assess their analysis needs and choose an appropriate time step to achieve the desired accuracy if taking a discrete time approach.

There are also limitations specific to our case example. First, in the application illustrated, we opted to use the GBD data for the baseline incidence and prevalence, given the limited age-specific incidence and prevalence data availability on anxiety disorders in Germany. It is important to recognize that the GBD data are estimates themselves, which are generated through the use of a Bayesian meta-regression tool on a substantially large number of periodically collected global data sources, allowing spatial and temporal extrapolation where there is data scarcity (16). If available, however, it would be more desirable to use age-specific data collected for a specific population of interest for improved projection accuracy. Second, the mortality risk ratio of anxiety disorders used for our projection was not age-specific or country-specific. We also assumed the same trend among men and women. While we think the assumption we made based on the empirical evidence is reasonable, the projection reliability can be expected to improve if age- and sex-specific data for a specific population of interest are available. This also speaks to the importance of increasing data availability in general and specifically in the area of mental health, which is also emphasized and integrated as an objective in the Comprehensive Mental Health Action Plan 2013–2030 of the World Health Organization (29). Third, we observed some irregularities in the remission probabilities estimated using the extended recursion formula. Mostly seen in the younger ages, these estimates that took values below zero were then omitted prior to modeling the remission probability. This occurrence may be attributable to possible inconsistencies in incidence and prevalence estimates we used; however, it is not verified and requires further investigation. Such irregularities, if encountered using other datasets, should be handled with caution. Fourth, we assumed that the prevalence of anxiety disorders among the German population and the migrants were similar. While there is some evidence to support this assumption (30), if the prevalence indeed differs between the two groups, then it would require a careful examination. Although a small impact of migration on prevalence has been shown in dementia under two extreme scenarios of all migrants are diseased and no migrants are diseased (9), this finding may not be generalizable to anxiety disorders as anxiety disorders occur in much younger ages where migration may be large, compared to dementia which is more relevant in older ages where migration may be small.

Our choice of a mental disorder for the illness-death model application is intentional. Mental disorders are highly prevalent globally and are a great public health concern. Modeling common mental disorders such as anxiety disorders with available data to facilitate prediction of future disease burden is of high public health relevance, especially at a time when we are seeing increased burden of mental disorders, yet still grapple with understanding the ultimate long-term impact of the COVID-19 pandemic on mental health (31–33).

The illness-death model provides a framework for chronic disease burden prediction and forms a basis for running simulations under varying assumptions in terms of transition probabilities. In the example shown here, the sex-specific estimates of the number of individuals with anxiety disorders over time from 2019 to 2030, can form a “base” population-level projection, where no additional factors influence transition probabilities in the model: incidence, remission, and mortality. Naturally, we would expect certain events and circumstances, such as the COVID-19 pandemic, may affect disease occurrence drastically. It is possible to incorporate such factors into the illness-death model and run simulations under varying incidence, mortality, or remission probabilities and additional assumptions as necessary. Such projections could allow for reasonable anticipation of a change in disease burden and population health needs over time and aid in preparation for resource allocation. Further applications of the illness-death model in areas where previous modeling attempts have been sparse therefore may provide valuable insight.

## Data Availability Statement

Publicly available datasets were analyzed in this study. This data can be found at: the GBD Results tool (http://ghdx.healthdata.org/gbd-results-tool) and the Federal Statistical Office (https://service.destatis.de/bevoelkerungspyramide/).

## Author Contributions

CI, TK, BTB, and RB made substantial contributions to the conception and design of the work. CI and RB collected data and ran the analysis. CI wrote the first draft of the manuscript. All authors contributed to the interpretation of the results, manuscript revision, as well as critical review, and approved the final version of the manuscript.

## Funding

We acknowledge financial support from the Open Access Publication Fund of Charité - Universitätsmedizin Berlin and the German Research Foundation (DFG).

## Conflict of Interest

Outside of the work presented in this paper, TK reports to have received personal compensation from Eli Lilly & Company, Teva Pharmaceuticals, TotalEnergies S.E., the BMJ, and Frontiers. Outside of the work presented in this paper, BTB reports to have received honoraria for roles including consultant, advisor, or CME speaker for the following entities: AstraZeneca, Bristol-Myers Squibb, Janssen, Lundbeck, Otsuka, and Servier. The remaining authors declare that the research was conducted in the absence of any commercial or financial relationships that could be construed as a potential conflict of interest.

## Publisher's Note

All claims expressed in this article are solely those of the authors and do not necessarily represent those of their affiliated organizations, or those of the publisher, the editors and the reviewers. Any product that may be evaluated in this article, or claim that may be made by its manufacturer, is not guaranteed or endorsed by the publisher.
