# Supplementary material for: Illness-Death Model as a Framework for Chronic Disease Burden Projection: Application to Mental Health Epidemiology
Source: Front Epidemiol. 2022 Jun 27;2:903652. doi: 10.3389/fepid.2022.903652 (PMC10910899; doi:10.3389/fepid.2022.903652)

Supplementary Material

1. **Supplementary Table 1. List of data sources used for projections of the prevalence of anxiety disorders**
2. **Supplementary Figure 1. Age-specific incidence, mortality, and remission probability of anxiety disorders among women, 2019**

Supplementary Table 1. List of data sources used for projections of the prevalence of anxiety disorders

Data source	**Global Burden of Disease Study 2019**	**Federal Statistical Office (Destatis): 14th Coordinated Population Projection for Germany**
Purpose	Prevalence, Incidence	Population count
Criteria used	Location: Germany<br>Year: 1990, 1991, 1992, 1993, 1994, 1995, 1996, 1997, 1998, 1999, 2000, 2001, 2002, 2003, 2004, 2005, 2006, 2007, 2008, 2009, 2010, 2011, 2012, 2013, 2014, 2015, 2016, 2017, 2018, 2019<br>Context: Cause<br>Age:1-4,5-9,10-14,15-19,20-24,25-29,30-34,35-39,40-44,45-49,50-54,55-59,60-64,65-69,70-74,75-79,80-84, 85-89, 90-94<br>Metric: number, percent, rate<br>Measure: prevalence, incidence<br>Sex: male, female, both<br>Cause: B.6.4 Anxiety disorders	Variant: 2<br>● Moderate development in fertility, life expectancy, and migration<br>● Fertility stabilizing at 1.55 children per woman<br>● Life expectancy at birth:<br>○ 2015/17: female - 83.2 years; male - 78.4 years<br>○ 2060: female – 88.1 years; male – 84.4 years<br>● Net migration (balance of immigration and migration):<br>○ 2018: 386,000 persons per year<br>○ 2026-2060: 206,000 persons per year
Link	VizHub - GBD Results (healthdata.org)	Bevölkerungspyramide: Altersstruktur Deutschlands von 1950 - 2060 (destatis.de)

Supplementary Figure 1. Age-specific incidence, mortality, and remission probability of anxiety disorders among women, 2019

A snapshot of anxiety disorders transition probabilities used in the illness-death model-based projection: a) age-specific incidence, b) mortality, and c) remission probability among women in Germany in 2019.

a)

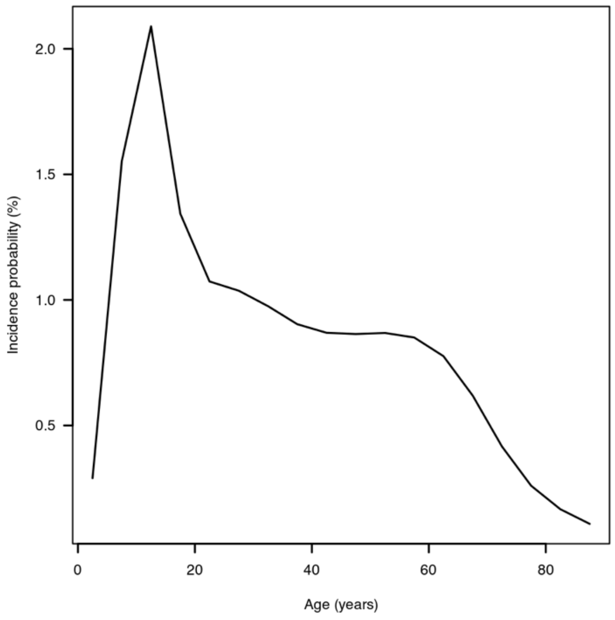

b)

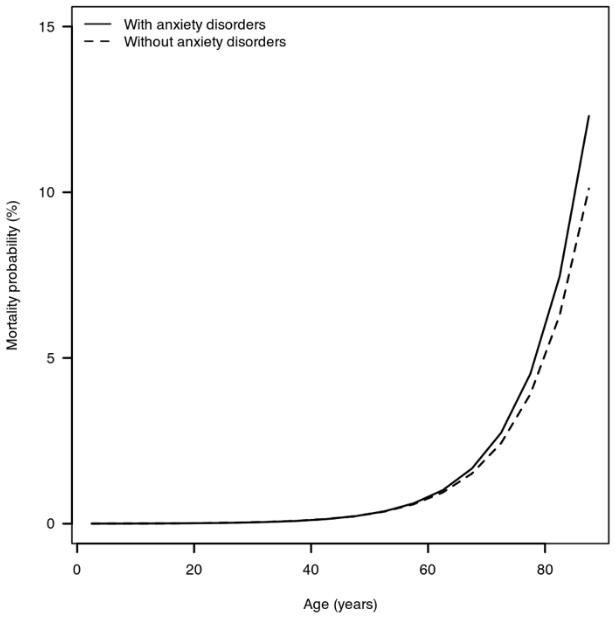

c)

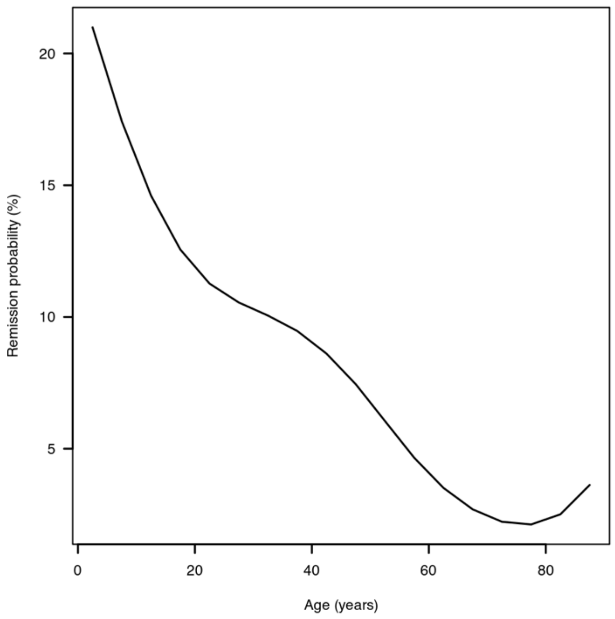

Supplement: Supplementary file 1 [file Data_Sheet_1.pdf]
